# Supplementary material for: Migration of Small Ribosomal Subunits on the 5′ Untranslated Regions of Capped Messenger RNA
Source: Int J Mol Sci. 2019 Sep 10;20(18):4464. doi: 10.3390/ijms20184464 (PMC6769788; doi:10.3390/ijms20184464)
Supplement: Supplementary file 1 [file ijms-20-04464-s001.pdf]

Supplementary Materials

for article

Migration of Small Ribosomal Subunits on the 5′ Untranslated Regions of Capped Messenger RNA

Nikolay E. Shirokikh, Yulia S. Dutikova, Maria A. Staroverova, Ross D. Hannan and Thomas Preiss

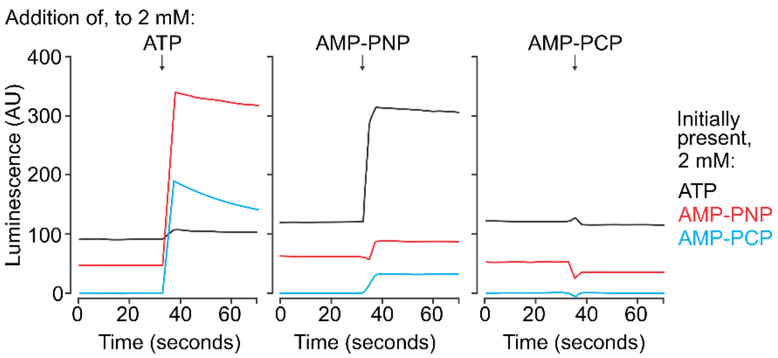

Supplementary Figure S1. In situ luminescence catalysed by purified firefly luciferase (Sigma-Aldrich, Saint Louis, MO, USA) added to reaction mixtures containing cell-free translation system based on mouse Krebs-2 cells lysate gel-filtered using Illustra MicroSpin G25 columns (GE Healthcare, Jefferson City, MO, USA), assembled generally as described in Section 2.1 main text and Materials and Methods. Addition of mRNA was omitted and either 2 mM of ATP (black line), 2 mM of AMP-PNP (red line) or 2 mM of AMP-PCP (blue line) was initially added during the assembly (see legend on right); prior to taking measurements the reaction mixtures were incubated at 30 °C for 2 min allowing equilibrium to settle and baseline to stabilise. Thirty-five seconds before the end of measurement, the mixtures were supplemented with 2 mM ATP (left), 2 mM AMP-PNP (middle) or 2 mM AMP-PCP (right; see labels on top). All nucleotide phosphates were added from a freshly prepared equimolar premix with magnesium acetate.

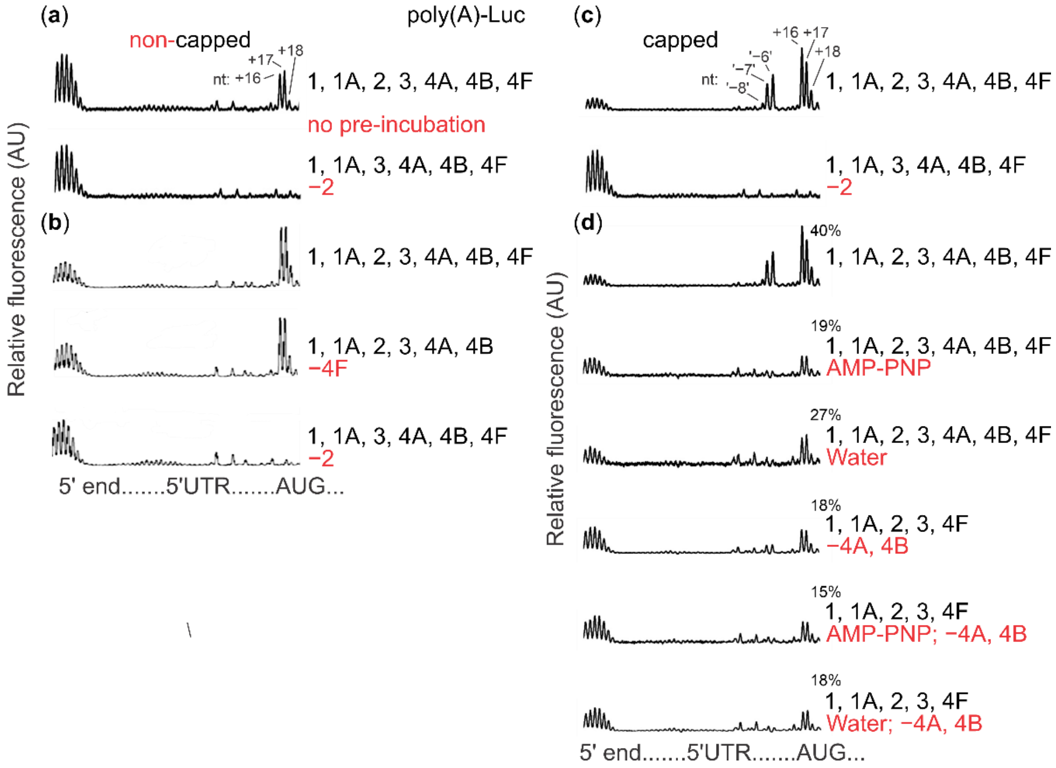

Supplementary Figure S2. Toeprint assay of ribosome:poly(A)-Luc (capped and noncapped) mRNA complexes (see Figure 4 legend for more details). (**a**,**b**) Reactions with noncapped poly(A)-Luc mRNA. (**c**.**d**) Reactions with capped poly(A)-Luc mRNA. (**a**) Original toeprinting conditions as described in [1,2]. eIFs and mRNA were not preincubated, and the reaction mixtures were assembled immediately to completion; compared to conditions employed elsewhere in this study, mRNA concentration was further lowered to 15 nM, and SSU concentration increased to 130 nM. eIF concentrations were as described in [1,2]. (**c**,**d**) Panels with the full factor sets and with/without eIFs 4A, 4B and ATP or AMP-

PNP represent independent repeats compared to the main text Figures 3, 4. ATP and AMP-PNP were added from freshly prepared equimolar premixes with magnesium acetate. Percent values indicate amounts of fluorescence (area under the curve) corresponding to all signals related to the cognate start codon of this mRNA (+16, +17, +18 nt peaks), relative to the total signal in the 5′UTR.

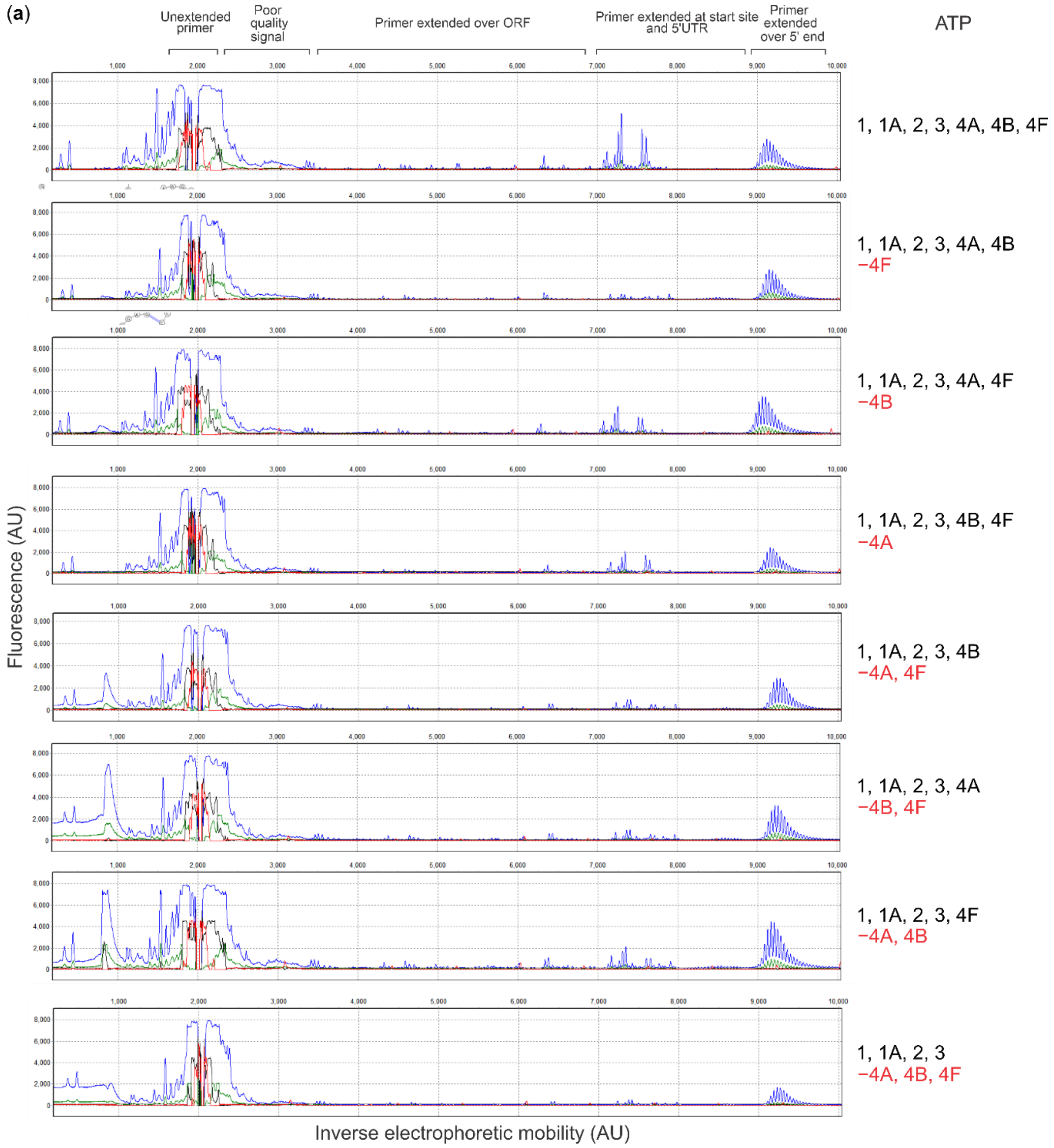

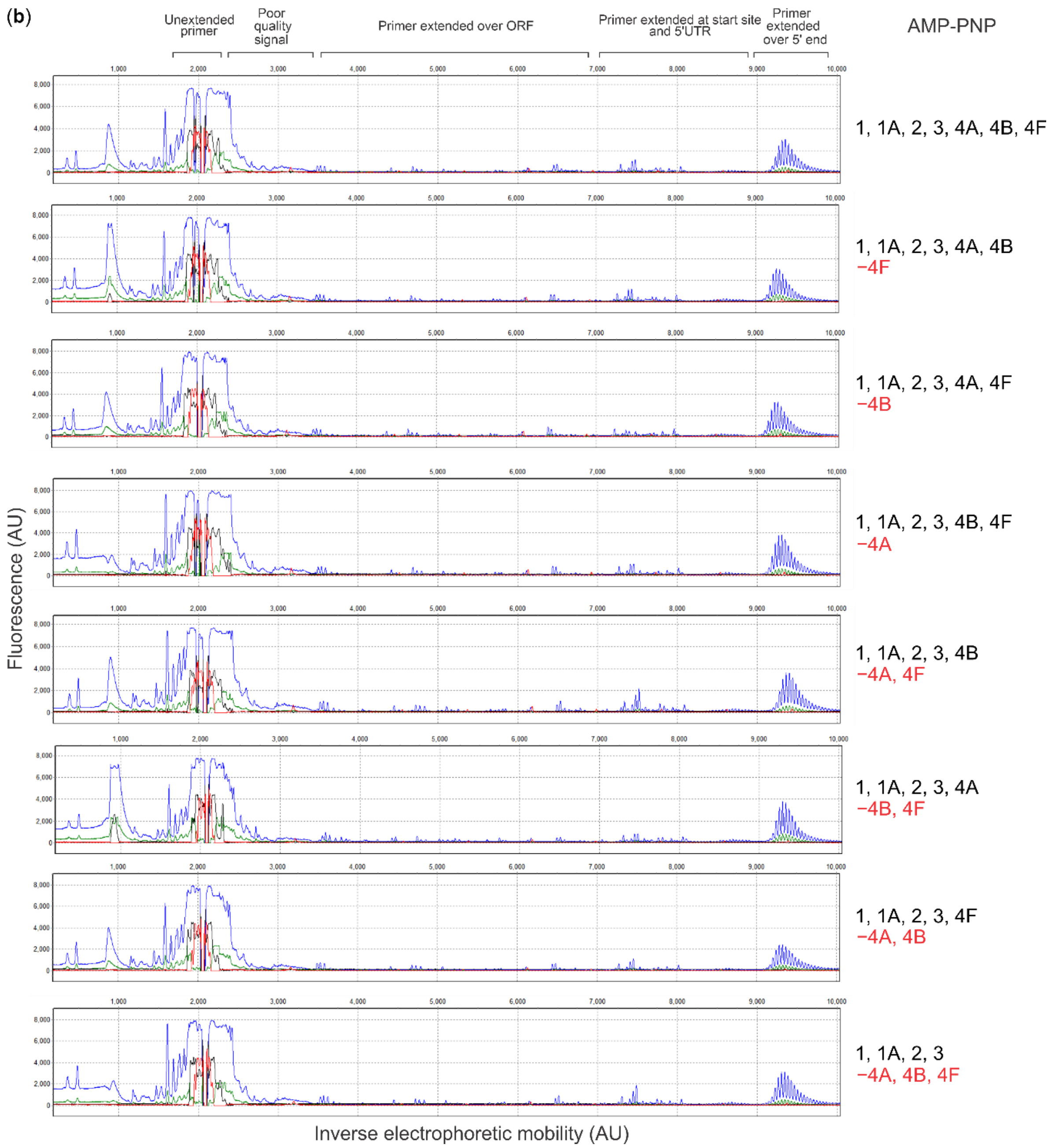
(b)
Unextended primer
Poor quality signal
Primer extended over ORF
Primer extended at start site and 5'UTR
Primer extended over 5' end
AMP-PNP
1, 1A, 2, 3, 4A, 4B, 4F
1, 1A, 2, 3, 4A, 4B
−4F
1, 1A, 2, 3, 4A, 4F
−4B
1, 1A, 2, 3, 4B, 4F
−4A
1, 1A, 2, 3, 4B
−4A, 4F
1, 1A, 2, 3, 4A
−4B, 4F
1, 1A, 2, 3, 4F
−4A, 4B
1, 1A, 2, 3
−4A, 4B, 4F
Fluorescence (AU)
Inverse electrophoretic mobility (AU)

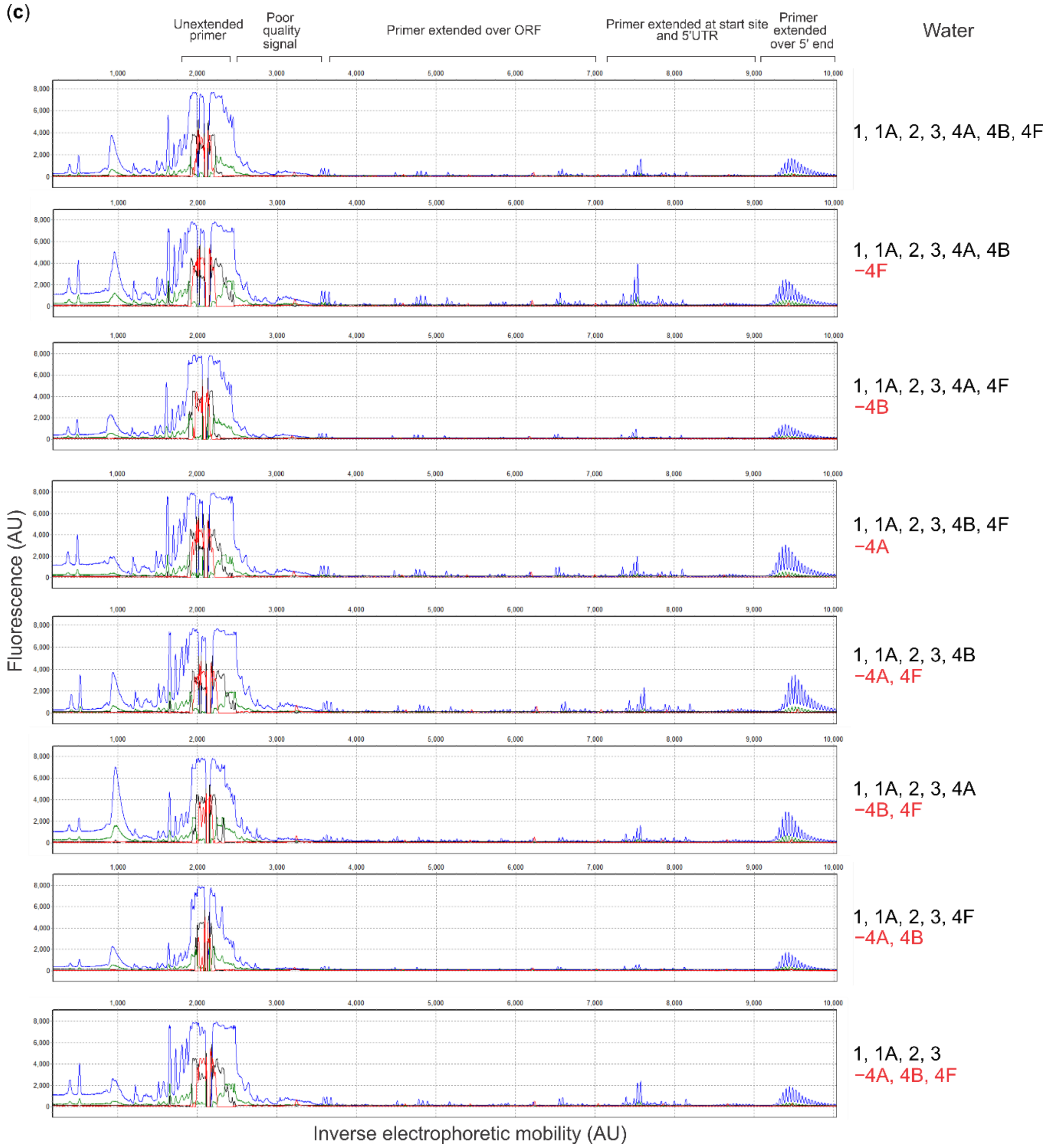

Supplementary Figure S3. Fluorescence of cDNA fragments generated in a reverse transcription reaction with ribosome:poly(A)-Luc (capped) mRNA complexes after electrophoretic separation (toeprint assay). Combinations of the initiation factors and nucleotide triphosphate or analogue used (ATP, (**a**); AMP-PNP, (**b**); none, (**c**)) are indicated on the right of each panel. See main text Figure 4, its legend and Materials and Methods for further experimental details. Presented are the original unprocessed traces; note that the processed and aligned cDNA traces in the Figures 4, 5 and Supplementary Figure S2 are flipped by the horizontal axis.

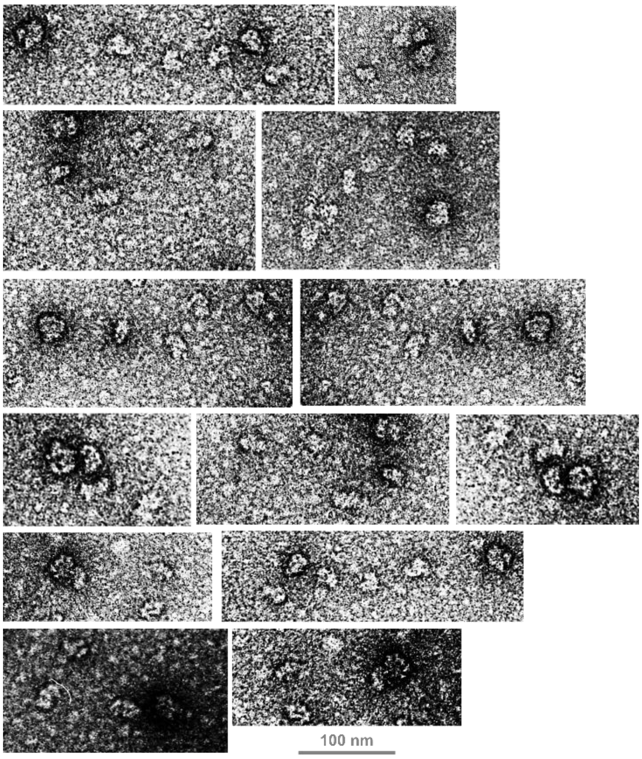

Supplementary Figure S4. High-contrast electron micrographs of mRNA:ribosomal complexes assembled in Krebs cells lysate on capped mRNA with LL1 5′UTR, from Figure 6 and other electron micrographs for similar samples. See Figure 6 legend and Materials and Methods for the description of sample preparation.

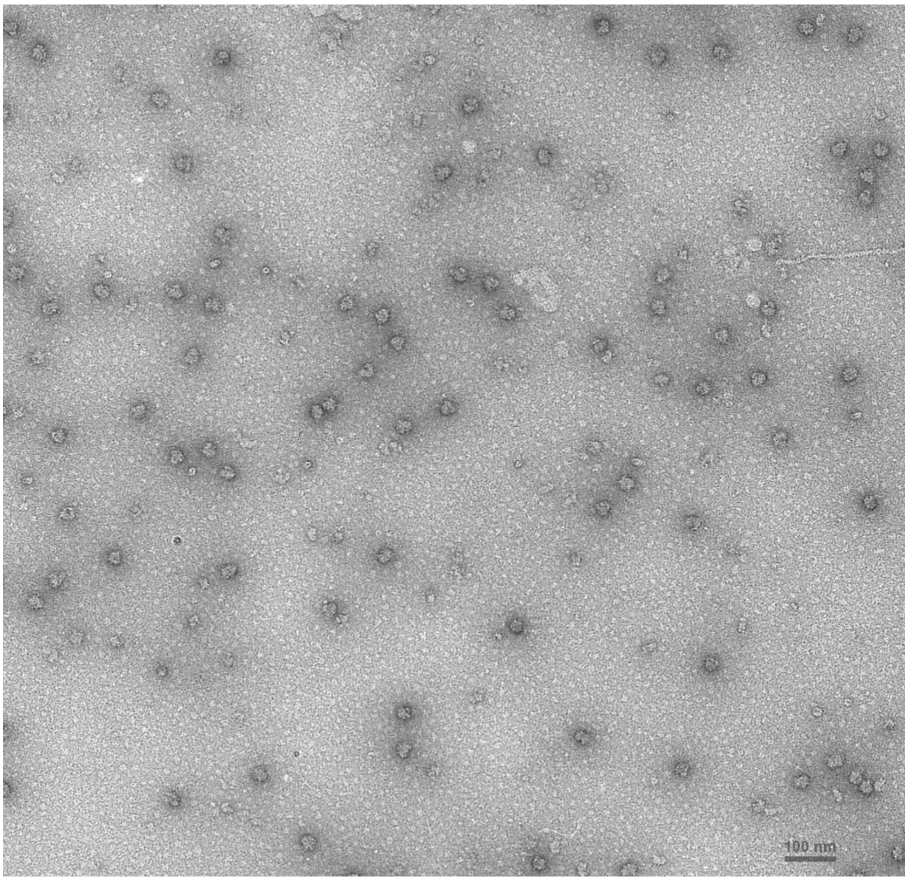

Supplementary Figure S5. Electron micrograph of mRNA:ribosomal complexes assembled in Krebs cells lysate on noncapped mRNA with LL1 5′UTR. See Figure 6 legend and Materials and Methods for the description of sample preparation; note that mRNA was uncapped and AMP-PNP:$Mg(OAc)_2$ was not added in this case.

References

1. Shirokikh, N.E.; Spirin, A.S. Poly(A) leader of eukaryotic mRNA bypasses the dependence of translation on initiation factors. *Proc. Natl. Acad. Sci. U.S.A.* **2008**, *105*, 10738-10743, doi:0804940105 10.1073/pnas.0804940105.
2. Shirokikh, N.E.; Alkalaeva, E.Z.; Vassilenko, K.S.; Afonina, Z.A.; Alekhina, O.M.; Kisselev, L.L.; Spirin, A.S. Quantitative analysis of ribosome-mRNA complexes at different translation stages. *Nucleic Acids Res.* **2010**, *38*, e15, doi:gkp1025 10.1093/nar/gkp1025.
